# Selective Detection of NADPH Oxidase in Polymorphonuclear Cells by Means of NAD(P)H-Based Fluorescence Lifetime Imaging

**DOI:** 10.1155/2008/602639

**Published:** 2008-10-19

**Authors:** R. Niesner, P. Narang, H. Spiecker, V. Andresen, K. -H. Gericke, M. Gunzer

**Affiliations:** ^1^Institute of Physical and Theoretical Chemistry, University Braunschweig, Hans-Sommer Straße 10, D-38106 Braunschweig, Germany; ^2^Helmholtz Centre for Infection Research, Inhoffenstraße 7, D-38124 Braunschweig, Germany; ^3^Centre for Immunology and Infection, Department of Biology, University of York, P.O. Box 373, York YO10 5YW, UK; ^4^LaVision Biotec GmbH, Meisenstr. 65, D-33607 Bielefeld, Germany; ^5^Institute of Molecular and Clinical Immunology, Otto von Guericke University Magdeburg, Leipzigerstrasse 44, D-39120 Magdeburg, Germany

## Abstract

NADPH oxidase (NOX2) is a multisubunit membrane-bound enzyme complex that, upon assembly in activated cells, catalyses the reduction of free oxygen to its superoxide anion, which further leads to reactive oxygen species (ROS) that are toxic to invading pathogens, for example, the fungus *Aspergillus fumigatus*. Polymorphonuclear cells (PMNs) employ both nonoxidative and oxidative mechanisms to clear this fungus from the lung. The oxidative mechanisms mainly depend on the proper assembly and function of NOX2. We identified for the first time the NAD(P)H-dependent enzymes involved in such oxidative mechanisms by means of biexponential NAD(P)H-fluorescence lifetime imaging (FLIM). A specific fluorescence lifetime of 3670±140 picoseconds as compared to 1870 picoseconds for NAD(P)H bound to mitochondrial enzymes could be associated with NADPH bound to oxidative enzymes in activated PMNs. Due to its predominance in PMNs and due to the use of selective activators and inhibitors, we strongly believe that this specific lifetime mainly originates from NOX2. Our experiments also revealed the high site specificity of the NOX2 assembly and, thus, of the ROS production as well as the dynamic nature of these phenomena. On the example of NADPH oxidase, we demonstrate the potential of NAD(P)H-based FLIM in selectively investigating enzymes during their cellular function.

## 1. Introduction

NADPH oxidase (NOX2) is a membrane-associated enzyme complex that is found on a variety of cells such as polymorphonuclear cells (PMNs, also neutrophil granulocytes), macrophages, and so forth [1]. These cells constitute the first cellular defence against microorganisms that have breached initial physical and chemical barriers of the innate immune system. Within peripheral tissues, such as the lung, these cells are able to recognise the invading pathogens through specific pattern recognition receptors on their surface [2]. The binding of the pathogens to these receptors initiates signalling pathways, which enable the cell to internalise the pathogen in a phagocytic vacuole or a phagosome, a process known as phagocytosis. Internalisation of the pathogen is followed by the production of reactive oxygen species (ROS), which are highly potent in killing the invading pathogens and, hence, constitute the major defence mechanism employed by the innate immune system [3]. ROS are synthesised in oxidative reactions based on the superoxide anion O_2_^−^, which in turn is the product of the following redox reaction catalysed by the activated NADPH oxidase:

(1) $$\text{NADPH}+{\text{O}}_{2}⇄\text{NADP}+{{\text{O}}_{2}}^{-}+{\text{H}}^{+}.$$

NOX2 is essentially made of a cytosolic protein-based heterotrimer (p40^phox^-p47^phox^-p67^phox^) and a membrane-bound protein-based heterodimer (gp91^phox^-p22^phox^), also known as cytochrome b_558_ [4]. Phosphorylation of the cytosolic subunits in an activated cell allows their translocation to the membrane-associated cytochrome b_558_, completing the assembly of the active NOX2 enzyme at the phagosome membrane. As a result, nicotinamide adenine dinucleotid phosphate (NADPH) is geometrically able to bind to NOX2, and the redox reaction for the production of O_2_^−^ can take place. More information about the structure of NOX2 and the mechanism of electron transfer within it can be found in [1, 5].

The importance of NOX2 and its role in the production of ROS are evident by individuals with genetic defects in one or more subunits of NOX2, a disorder known as chronic granulomatous disease (CGD). These patients have a higher susceptibility to bacterial and fungal infections owing to defects in the proper assembly and function of this enzyme. One such pathogen of clinical importance is *Aspergillus fumigatus*, a ubiquitous fungus that reproduces asexually by the production of hydrophobic spores (conidia). The conidia can be inhaled in large numbers due to their small diameter of 2-3 *μ*m [6]. However, they rarely cause problems in healthy individuals due to an effective defence provided by resident lung alveolary macrophages (AMs), which also kill conidia in an ROS-dependent manner [7]. Conidia that escape AMs start germinating into hyphae, which are able to invade tissues. However, hyphae are controlled by PMNs [8], which can bring about their death by a combination of nonoxidative and ROS-dependent mechanisms. Consequently, invasion of tissues and subsequent spread of the fungus to distant organs requires significant immunosupression, as seen in bone marrow transplant recipients. Invasive aspergillus infections are, therefore, a significant problem for these patients and for CGD patients, since they are unable to effectively eliminate the fungal conidia and hyphae.

Although ROS have been shown to be indirectly involved in the defence against pathogens like *A. fumigatus*, the exact subcellular location where they are produced is uncertain [7]. Furthermore, the dynamics of ROS production with respect to host-pathogen interactions has not been previously investigated to our knowledge. Due to the central role of NADPH oxidase in the oxidative mechanisms against invading pathogens, approaches to identify the location and to monitor the dynamics of ROS production based on the visualisation of NADPH oxidase in cells, are particularly appropriate.

Biexponential fluorescence lifetime imaging (FLIM) based on the fluorescence of the coenzymes NADH (nicotinamide adenine dinucleotide) and NADPH (nicotinamide adenine dinucleotide phosphate), hereafter NAD(P)H, has already been extensively employed in biosciences and biomedicine to differentiate between free NAD(P)H and enzyme-bound NAD(P)H, that is, NAD(P)H which participates in metabolic processes, within living cells and, thus, to monitor the cellular redox metabolism [9–14]. The method relies on the different fluorescence lifetimes of free (approx 400 picoseconds) and enzyme-bound NAD(P)H (approx. 2000 picoseconds) [12, 15]. The oxidised forms NAD(P) of these coenzymes are practically nonfluorescent, that is, their fluorescence quantum yield is significantly lower than that of the reduced forms.

While differences in the fluorescence lifetimes of the free coenzymes (NADH/NADPH, folded/unfolded structure) are rather small [16], the fluorescence lifetime of the enzyme-bound NAD(P)H is dramatically influenced by the enzyme to which NAD(P)H is bound to, as demonstrated in detailed extracellular time-resolved experiments [17–19]. Also intracellular FLIM results indicate the presence of enzyme-NAD(P)H complexes characterised by different fluorescence lifetimes within cells: the histogram of the fluorescence lifetimes of the enzyme-bound NAD(P)H has often not the shape and the width expected if considering only the effect of noise on the evaluation technique and the limitations of the experimental setup [9, 20, 21]. A selective lifetime-based identification of enzyme-NAD(P)H complexes in cells, however, has not been reported to our knowledge.

In this work, we determine the specific fluorescence lifetime of NADPH when bound to enzymes responsible for oxidative processes following phagocytosis, that is, in PMNs mainly NADPH oxidase, and identify the activated (assembled) NADPH oxidase in PMNs by means of NAD(P)H-based biexponential FLIM. Since it is well known that the fluorescence lifetime is significantly influenced by the environment of the chromophore (in our case, NADPH), for selectively detecting the NADPH-NOX2 complex in cells, we choose to perform comparative FLIM experiments on unstimulated murine PMNs and on PMNs treated with specific activators (phorbol 12-myristate 13-acetate) and inhibitors (4-(2-aminoethyle)-benzenesulfonyl) of NOX2 rather than comparing the fluorescence lifetime of the NADPH-NOX2-complex under extra- and intracellular conditions, respectively. A further reason for this choice is that the extracellular activation of NADPH oxidase is difficult as well as uncertain due to the complex structure and function of the enzyme within the cell [22].

Moreover, we localise the NADPH-NOX2-complex in PMNs during interaction with conidia and hyphae of *Aspergillus fumigatus* and, thus, identify the regions in a cell where ROS are produced during cellular response to the pathogen. Finally, the use of a time-gated multifocal fluorescence lifetime imaging device, which enables relatively fast FLIM (up to 1 FLIM-frame/minute for NAD(P)H fluorescence), allowed us to observe not only where but also when NOX2 is activated (ROS are produced) in PMNs interacting with aspergillus hyphae.

FLIM is a microscopy technique, which, as the name suggests, records the fluorescence lifetime of the sample in a spatially resolved manner. Due to the fact that the fluorescence lifetime of chromophores is influenced by environmental as well as molecular parameters, this method is particularly appropriate to monitor modifications in biological systems as demonstrated by a large number of FLIM applications. Different FLIM techniques have been developed and used for this purpose, for example, frequency-domain techniques [23–26], time-correlated single-photon counting techniques [27–30], techniques based on time-gating [31–35] or on the use of streak cameras [36]. While the advantages and disadvantages of these techniques have been reviewed in detail elsewhere [23, 27, 35], inhere we explain only the reason for the selection of a time-gated camera-based multifocal FLIM technique in our experiments. Although our method is very sensitive to signal and background noise and uses only a small fraction of the total fluorescence signal as compared to, for example, TCSPC techniques, it is one of the fastest FLIM procedures due to the possibility to scan the sample with up to 64 beams simultaneously, that is, a data acquisition speedup of 64×.

## 2. Materials and Methods

### 2.1. Time-Resolved Two-Photon Laser Scanning Microscopy

All experiments were carried out using a specialised two-photon laser-scanning microscope (TPLSM) based on a tunable (720 to 920 nm) Ti:Sa laser system (MaiTai, Spectra Physics, Darmstadt, Germany) and on a commercial scan-head (TriMScope, LaVision BioTec, Bielefeld, Germany), which allows multibeam (2, 4, … to 64 beamlets) scanning of the sample without cross-talk between neighboring beamlets, that is, interference free and without loss in spatial resolution [37, 38]. The multibeam scanning permits a speedup of data acquisition proportional to the splitting of the main laser beam. The excitation beam(s) is focused into the sample by a 20× objective lens with NA = 0.95 and working distance of 2 mm (Olympus, Hamburg, Germany).

In steady-state experiments, we used a CCD camera Sensicam QE (LaVision, Goettingen, Germany) at dwell times of 0.4 microsecond to 1 microsecond per pixel (332 × 332  nm^2^) as detection unit. The spatial resolution is 330 nm lateral and 1.36 *μ*m axial measured on yellow-green fluorescing microbeads (100 nm diameter) at 800 nm excitation wavelength [37]. In FLIM experiments, the fluorescence signal was detected using an intensified CCD camera with variable time gate (200–1000 picoseconds) (PicoStar, LaVision) at dwell times of 1 microsecond to 5 microseconds per pixel (350 × 350  nm^2^). Under these conditions, we measured a slightly lower lateral resolution (550 nm) than in steady-state experiments at the same axial resolution. The time-gate for all NAD(P)H-FLIM experiments was 400 picoseconds, the time step between two data points was chosen between 100 and 400 picoseconds and the total measuring time varied between 4 and 6 nanoseconds. Typically, we used 21 time gates at an acquisition time of 1 to 3 seconds per gate in order to perform the biexponential fit of the NAD(P)H decay curve. The best attainable time resolution of our setup measured under almost noise-free conditions was 9 picoseconds and corresponds to the jitter of the time-gated device [39]. The laser power was varied between 1 and 7 mW per beamlet both in steady-state and in time-resolved experiments. The spectral selection of exogenous and endogenous chromophores was made by means of interference filters (D or HQ from AHF, Tuebingen, Germany).

### 2.2. Principle of Time-Gating FLIM

Time-gating FLIM measures in a spatially resolved manner the fluorescence decay curve of a sample in time domain, that is, records the fluorescence decay curve in each pixel of an image. Thereby, for each data point on the decay curve, only the fluorescence signal, which occurs within a small time period (time gate) at a certain time point with respect to the laser pulse, is measured. In order to obtain the complete fluorescence decay curve, the position of the time gate is shifted from the reference laser pulse to (maximally) the subsequent pulse. Thus this method necessitates an accurate synchronizsation of the gating controller with the pulse train of the excitation laser.

### 2.3. Evaluation of the FLIM Data

In order to extract the fluorescence decay time of the free and enzyme-bound NAD(P)H, respectively, as well as their contribution to the total fluorescence signal from the measured fluorescence decay curves, we performed biexponential approximations using both the standard gradient method (Levenberg-Marquardt) and a fast noniterative evaluation method described elsewhere [20, 40]. Both techniques led to similar results within the error margins. The advantages of the noniterative analysis against the gradient method are its computational speed and the necessity for less data points on the fluorescence decay curve, that is, speedup of data acquisition [20]. Its major limitation is the instability to signal noise [40].

In order to reduce the effect of signal and background noise on the data, we performed a 3 × 3 pixel Gauss convolution on all intensity images used for FLIM evaluation. This intermediate step leads to loss of spatial information but increases the accuracy of the fluorescence lifetime results.

### 2.4. Isolation of Polymorphonuclear Cells (PMNs)

PMNs were isolated from the bone marrow of Balb/c mice (Harlan, Germany) as described earlier [41]. Briefly, bone marrow cells were flushed from tibiae and femurs of mice with phosphate buffer solution (PBS) supplemented with 1% fetal calf serum (FCS). Following erythrocyte lysis, the cell suspension was filtered through a 100 *μ*m nylon stainer to remove tissue fragments. To avoid nonspecific binding, the cells were then incubated with an Fc*γ*RIII/II antibody/Fc-block (BD Biosciences) at 0.5 *μ*mol/mL on ice for 15 minutes, washed and subjected to positive selection using Gr1-labeled magnetic particles (clone RB6-8C5, BD Biosciences) in BDIMag buffer. 50 *μ*L beads were added for every 10^7^ cells, which were then kept on ice for 30 minutes. All the cells bound to the magnet were retrieved and washed. The cells were counted and kept in PBS + 1% FCS on ice until use. All animals were housed in specific pathogen free (SPF) conditions at Helmholtz Centre for Infection Research, and animal experiments were carried out in accordance with the national and institutional guidelines.

### 2.5. Fungus *Aspergillus Fumigatus*

*Aspergillus fumigatus* (strain D141) was kindly provided by Dr. Axel Brakhage (Hans Knoll Institute, Jena, Germany). The fungus was cultivated and harvested as described earlier [41]. Briefly, *A. fumigatus* was grown on aspergillus minimal media (AMM) agar plates at 37° for 5 days. AMM was supplemented with 1% (w/v) glucose, trace elements (a mixture of FeSO_4_, MnSO_4_, ZnSO_4_, CuSO_4_ and NaB_2_O_7_), and a combination of Penicillin and Streptomycin (100 U/mL). Conidia suspensions were prepared by flooding the agar plates with distilled water, gently scraping the mycelial mat with an inoculation loop, and resuspending it in sterile water. The suspensions were then filtered through a 100 *μ*m nylon cell strainer (BD Falcon) to remove hyphal fragments, and stored at 4°C until further use. To obtain aspergillus hyphae, viable conidia at a concentration of 10^7^/mL were incubated at 37°C in RPMI supplemented with 5% FCS for 5 hours with constant shaking at 180 rpm. The resulting germ tubes were washed once in PBS and used immediately for imaging experiments.

### 2.6. Staining; Activation/Inhibition of NOX2

For steady-state experiments, the PMNs and the fungus were stained with cell tracker orange (CTO) (Invitrogen, Goettingen, Germany) and with fluoresceine isothiocyanate (FITC) (Invitrogen), respectively. The cells or the fungus were resuspended in PBS at a concentration of 10^7^/mL. FITC at a concentration of 0.15 mg/mL and CTO at a concentration of 5 *μ*g/mL were added to the fungus and the cells, respectively. The suspension was kept at 37°C for 20 minutes, following which the cells and the fungus were washed to remove all traces of free dye. For FLIM measurements, the cells and the fungus were not stained prior to measurements.

For the activation and inhibition of the NADPH oxidase in PMNs, we used phorbol 12-myristate 13-acetate (PMA, Fluka, Taufkirchen, Germany) at a concentration of 30 nmol/L and 4-(2-aminoethyle)-benzenesulfonyl (AEBSF, Sigma Aldrich) at a concentration of 5 *μ*mol/L, respectively. Phosphate buffer solution (PBS) and PBS + 1% fetal calf serum (FCS) were used as cell media. All biological samples were tempered during the experiments at 37°C by means of a temperature-controlled microscope stage.

## 3. Results

### 3.1. Phagocytosis Studied by TPLSM

In preliminary steady-state TPLSM experiments, we 3D imaged PMNs phagocyting conidia and hyphae of *Aspergillus fumigatus*. These experiments confirmed previous results on phagocytosis [41] and ensured that at laser powers of 1–7 mW/beamlet, at which both the steady-state and the time-resolved experiments were performed, the function and morphology of cells are not injured.

**Figure 1:**
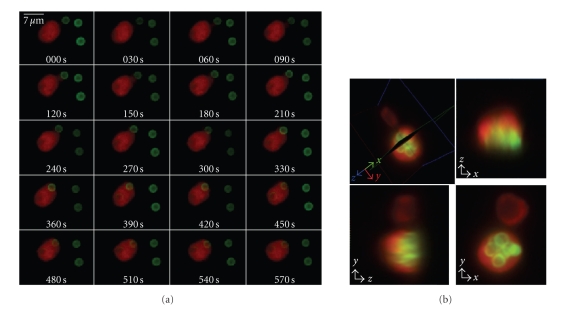
(a) Time series of 2D fluorescence images obtained by means of steady-state TPLSM representing a neutrophil granulocyte (PMN) phagocyting one of three conidia of *Aspergillus fumigatus*. The fluorescence of the CTO stained PMN (red) was observed at 590 nm, while that of the FITC stained fungus (green) was observed at 535 nm. (*λ*_exc_ = 800  nm, time step = 30 seconds, time lapse = 10 minutes) (b) 3D fluorescence image of a PMN and phagocytosed conidia. TPLSM results reveal that the fungal spores are readily internalised by the PMNs. The *z*-step between two consecutive images is 1 *μ*m.

As illustrated in Figure 1(a) and in supplementary movie 1 available online at doi:10.1155/2008/602639, our experiments demonstrate the dynamic nature of interactions between PMNs and both aspergillus hyphae (ribbon-shaped) and conidia (small, round), ranging from intensive contacts to uptake. While the conidia are readily internalised by PMNs (Figure 1(b)), the hyphae being much larger in size can be seen interacting with several PMNs all along their surface (supplementary movie 1). These observation corroborate our previous results on phagocytosis of *A. fumigatus* by PMNs obtained by means of wide field microscopy [41].

CTO-stained PMNs (red) and the FITC-stained fungus (green) were excited at 800 nm. The CTO emission was observed at 590 nm (interference filter D 590/50), whereas the FITC emission was detected at 535 nm (interference filter D 535/50). The time step between two consecutive 2D fluorescence images was 30 seconds, while that between consecutive 3D fluorescence stacks was 60 seconds.

Furthermore, it is well known that phagocytosed conidia within the phagosomes as well as hyphae in contact with PMNs initiate the assembly of NADPH oxidase in the cells on different pathways depending on the pattern recognition receptor that binds to the fungus. This results in the production of ROS either within the phagosome, directly attacking the internalised conidium, or, extracellularly, where the cell interacts with hyphae.

If no stimulus is present, the two parts of NOX2 are disjoined and the enzyme is inactive, that is, it has no catalytic function. Stimuli, for example, PMA as activating substance [42] or pathogens like the considered fungus, induce the binding of the cytosolic trimer p40^phox^-p47^phox^-p67^phox^ to the dimmer gp91^phox^-p22^phox^ and make the catalysis of the redox reaction (1) possible, that is, NADPH is geometrically able to bind to NOX2. The catalytic function of NOX2 can be inhibited by adding AEBSF, which binds to the dimer gp91^phox^-p22^phox^ and, thus, prevents the cytosolic trimer to bind to the latter in a competitive reaction [43]. Only when NADPH binds to the NADPH oxidase, the enzyme is visible by means of NAD(P)H-based fluorescence microscopy.

### 3.2. Steady-State and Time-Resolved Imaging Based on NAD(P)H Fluorescence

The autofluorescence of PMNs, like that of most animal cells, mainly originates from the reduced forms of NAD(P)H, if the cells are excited at approximately 750 nm. The main interfering fluorescence signal stems from flavoproteins. However, if observing the emitted light at approximately 450 nm, an accurate spectral selection of NAD(P)H is achieved [44].

**Figure 2:**
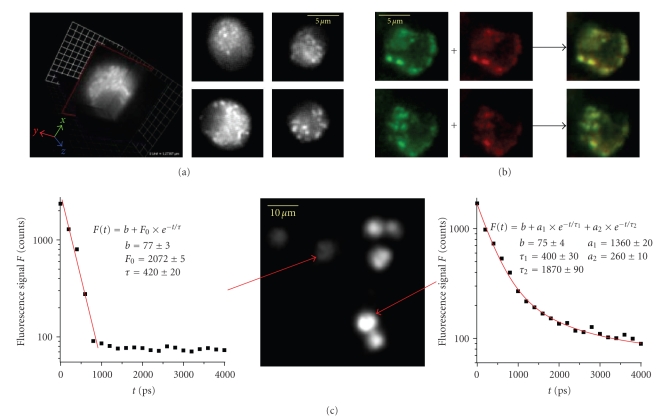
(a) 3D and 2D images obtained by means of steady-state TPLSM showing the endogenous fluorescence of PMNs. The endogenous fluorescence intensity is low in the nuclei and high in small organelles around the nucleus supposed to be mitochondria. (b) Endogenous fluorescence (green, left) and Rhodamine 123 fluorescence (red, centre) are shown to be colocalised in living PMNs (yellow, right). Since Rhodamine 123 accumulates in the mitochondria of living cells, we can conclude that the bright structures in the autofluorescence image are mithocondria. (c) Fluorescence decay curves in different PMNs 10 minutes after NaCN addition (30 *μ*mol/L). While in some cells (left diagram) the decay is monoexponential, that is, only free NAD(P)H, in other cells (right diagram), a biexponential behaviour of the decay has been retrieved, that is, both free and enzyme-bound NAD(P)H is monitored. These results indicate that under the given experimental conditions (*λ*_exc_ = 760  nm and fluorescence detection at 460 nm), the endogenous cellular fluorescence mainly originates from the coenzymes NADH and NADPH.

In our experiments, several observations suggest that NAD(P)H is responsible for the autofluorescence of PMNs excited at 760 nm and observed at 460 nm (interference filter D 460/50). First, the PMNs' fluorescence mainly originates from small structures around the nucleus and is very low within the nuclei (Figure 2(a)). These small bright structures were confirmed to be mitochondria in colocalisation experiments of autofluorescence and Rhodamine 123 fluorescence (Figure 2(b)). Rhodamine 123 specifically accumulates in mitochondria of living cells [45]. Furthermore, we observed an increase of the total endogenous fluorescence immediately after adding NaCN (30 *μ*mol/L in cell suspension), typical for NAD(P)H fluorescence [9, 44]. 10 minutes after NaCN addition, time-resolved experiments revealed that in some cells the endogenous fluorescence decay is monoexponential (decay time ≈400 picoseconds), while in other cells it can be well approximated by a biexponential function (decay times ≈400 picoseconds and ≈1900 picoseconds, resp.) mainly dominated by the short fluorescence lifetime term (Figure 2(c)). Since it is well known that free NAD(P)H has a fluorescence lifetime of approximately 400 picoseconds whereas enzyme-bound NAD(P)H has a longer fluorescence lifetime in nanosecond range, we conclude that the cells characterised by a monoexponential autofluorescence decay contain only free NAD(P)H and are dead, while the other cells contain both free and enzyme-bound NAD(P)H, are, however, characterised by a low-metabolic activity. Similar observations have already been reported [9].

**Figure 3:**
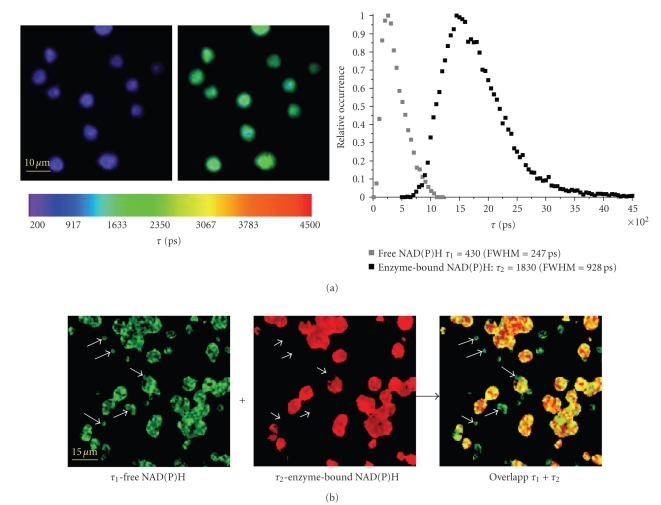
(a) Fluorescence lifetime images of free (*τ*_1_ image) and enzyme-bound NAD(P)H (*τ*_2_ image), respectively, as they resulted from the biexponential FLIM experiments performed on PMNs suspended in PBS + 1% FCS. The distributions of *τ*_1_ and *τ*_2_, respectively, in these images are shown in the left diagram. The fluorescence lifetime images were obtained by overlapping intensity images in gray scale with the spatially resolved fluorescence lifetime information in colour scale. (b) *τ*_1_ image (green) and *τ*_2_ image (red) obtained in FLIM experiments on a mixed suspension of PMNs and aspergillus conidia, as well as, the overlapp of the *τ*_1_ and *τ*_2_ images. The overlapped image confirms that conidia contain only free NAD(P)H, that is, their metabolic activity is very low, whereas PMNs contain both free and enzyme-bound NAD(P)H.

Biexponential FLIM experiments allow us to differentiate in each pixel of the considered image between free NAD(P)H and NAD(P)H, which is involved in cellular redox reactions catalysed by the enzymes, to which the coenzyme is bound to (Figure 3(a)). The fluorescence decay curve *F*(*t*) in each pixel is fitted by

(2) $$F(t)=a_{1}\cdot e^{-t/\tau_{1}}+a_{2}\cdot e^{-t/\tau_{2}}+\varepsilon .$$

The indices 1 and 2 correspond to the free and to the enzyme-bound NAD(P)H, respectively. *ε* is the background, *τ* the fluorescence lifetime, and *a* the prefactor corresponding to the concentration of the chromophore.

The average fluorescence lifetime of free NAD(P)H in murine PMNs amounts to 430 ± 40 picoseconds, while that of the enzyme-bound NAD(P)H is 1870 ±30 picoseconds (statistics over 140 cells). The fluorescence lifetime of free NAD(P)H measured in PMNs well agrees with the average lifetime over the folded and unfolded states of NADH (440 ± 15 picoseconds) and NADPH (410 ± 15 picoseconds) measured in solution. The fact that the fluorescence lifetime of the enzyme-bound NAD(P)H strongly depends on the partner enzyme is reflected in the wide distribution of bound NAD(P)H lifetimes (FWHM of approx. 1000 picoseconds) as compared to that of free NAD(P)H lifetimes (FWHM of approx. 250 picoseconds).

A further result of the biexponential FLIM experiments based on NAD(P)H fluorescence in PMNs is the ratio *a*_2_·*τ*_2_/(*a*_1_·*τ*_1_ + *a*_2_·*τ*_2_) image, that is, the contribution of the enzyme-bound NAD(P)H to the total fluorescence signal. Under the given experimental conditions (PBS + 1% FCS suspension and 37°C) the average ratio *a*_2_·*τ*_2_/(*a*_1_·*τ*_1_ + *a*_2_·*τ*_2_) amounts to 94 ± 2.3%, which indicates a high-metabolic activity of the cells.

NAD(P)H-based FLIM experiments performed on conidia in PBS revealed that within the first 2 hours after removing them from 4°C, the spores mainly contain free NAD(P)H with a typical lifetime of 440 ± 35 picoseconds (statistics over 200 conidia).

FLIM measurements on mixed suspensions of PMNs and conidia demonstrate that the very low-metabolic activity of the latter can easily be used to identify the spores in an NAD(P)H-based FLIM image. As illustrated in Figure 3(b), both PMNs and conidia can be seen in the *τ*_1_-image of free NAD(P)H, whereas only the cells appear in the *τ*_2_-image of enzyme-bound NAD(P)H.

 NAD(P)H-based FLIM experiments on aspergillus hyphae led to similar results as those on PMNs. The average ratio *a*_2_·*τ*_2_/(*a*_1_·*τ*_1_ + *a*_2_·*τ*_2_) amounts to 92 ± 3%, the average lifetime of the enzyme-bound NAD(P)H to 1820 ± 160 picoseconds and that of the free NAD(P)H to 420 ± 40 picoseconds (statistics over 72 hyphae).

The FLIM images shown in this and in the following sections except for those shown in Figure 3(b) were obtained by overlapping fluorescence intensity images in gray tones with the corresponding lifetime image (colour scale) in order to better identify correlations between cellular structures and fluorescence lifetimes. The corresponding colour scales are shown in each figure.

### 3.3. Activation and Inhibition of the NADPH Oxidase in PMNs

The main objective of this work is to identify the NAD(P)H-dependent enzymes involved in the oxidative mechanisms following phagocytosis from the pool of cellular mitochondrial and nonmitochondrial NAD(P)H-dependent enzymes by determining the specific fluorescence lifetime of NADPH when bound to the oxidative enzymes and, thus, to localise these enzymes in PMNs by means of NAD(P)H-based FLIM. Since the NADPH oxidase is the predominant enzyme responsible for ROS production (oxidative mechanisms) in PMNs, we assume that in these cells a specific fluorescence lifetime of NAD(P)H bound to oxidative enzymes mainly originates from the NADPH oxidase.

For this purpose, we performed comparative NAD(P)H-based FLIM experiments on PBS + 1%FCS suspensions of PMNs, to which we added either PMA as a specific NOX2 activator (activator for oxidative processes) or AEBSF as a specific NOX2 inhibitor or AEBSF followed by PMA as a negative control of the stimulation.

**Figure 4:**
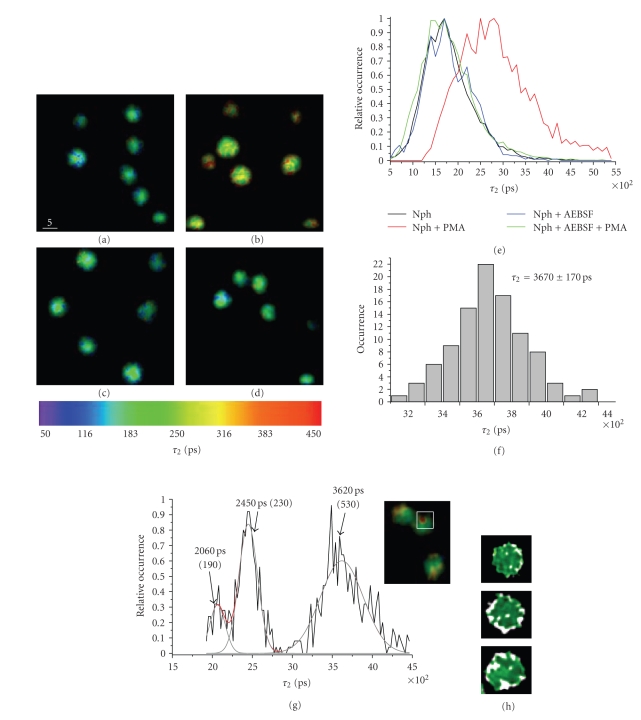
Fluorescence lifetime images of enzyme-bound NAD(P)H (*τ*_2_ images) in naive PMNs (a), in PMNs after addition of PMA (NADPH oxidase activator) (b), in PMNs after addition of AEBSF (NADPH oxidase inhibitor) (c), and in PMNs after addition of AEBSF followed by PMA as a negative control of the stimulation (d). Note that the fluorescence lifetime after addition of PMA (Figure 4(b))—especially in the membrane regions (Figure 4(h))—is longer than in the other images (Figures 4(a), 4(c), 4(d)). The increased average fluorescence lifetime after the stimulation with PMA is confirmed by the corresponding *τ*_2_-histograms in (e). Statistics over regions with increased *τ*_2_ in PMNs treated with PMA allowed a quantification of the fluorescence lifetime corresponding to the NADPH bound to NADPH oxidase (Figure 4(f)). The *τ*_2_-distribution in each region of increased fluorescence lifetime in PMNs treated with PMA was evaluated in order to identify the specific fluorescence lifetime peak of NADPH bound to NADPH oxidase as shown in (g). Figure 4(h) depicts the overlapp of *τ*_2_-maps of PMNs activated with PMA (green) and of regions of NOX2-specific fluorescence lifetime (3670±170 picoseconds) in the same cells (white). All fluorescence lifetime images were obtained by overlapping intensity images in gray scale with the spatially resolved fluorescence lifetime information in colour scale.

In these measurements, both the fluorescence lifetime of the free NAD(P)H and the ratio *a*_2_·*τ*_2_/(*a*_1_·*τ*_1_ + *a*_2_·*τ*_2_) do not significantly change on addition of either PMA or AEBSF or AEBSF followed by PMA as compared to untreated murine cells (data not shown). The fluorescence lifetime *τ*_2_ of the enzyme-bound NAD(P)H remains the same as in untreated cells on addition of AEBSF and of AEBSF followed by PMA (Figures 4(a), 4(b), 4(d)) but dramatically increases on addition of PMA (Figure 4(c)). The following average fluorescence lifetimes *τ*_2_ have been measured: 1870 ± 30 picoseconds (140 murine cells), 1860 ± 45 picoseconds on addition of AEBSF (115 cells), and 1840 ± 50 picoseconds on addition of AEBSF followed by PMA (108 cells). The addition of PMA resulted in significantly larger *τ*_2_-values, which strongly varied between 2140 picoseconds and 3080 picoseconds (90 cells).

We observed that the increase in the fluorescence lifetime of the enzyme-bound NAD(P)H in PMNs treated with PMA primarily appears in the membrane regions. On time (after some tens minutes in the presence of stimulus), the increased fluorescence lifetime extends over the whole cell. In order to quantify the shift in the *τ*_2_-histograms measured in PMNs treated with PMA as compared to those of untreated cells, we considered rectangular regions in the cell, which showed an increased fluorescence lifetime and made corresponding *τ*_2_-histograms. Such histograms could be well approximated by multipeak Gauss distributions (Figure 4(g)). The analysis of the *τ*_2_-histograms of 98 regions of increased fluorescence lifetime in PMNs treated with PMA repeatedly showed a peak at approximately 3600 picoseconds (Figure 4(f)). An average fluorescence lifetime *τ*_2_ of 3670 ± 170 picoseconds specific for these regions of increased fluorescence lifetime in PMNs treated with PMA was determined (Figure 4(f), statistics over 98 regions). Similar analysis of regions in PMNs alone as well as in PMNs treated with AEBSF or with AEBSF followed by PMA showed that this peak at approximately 3650 picoseconds is specific only for cells treated with PMA. Since neither PMA nor ROS nor any part of NOX2 are expected to fluoresce under the given conditions and the main function of PMA in PMNs is the activation of NOX2, we assume that the determined fluorescence lifetime of approximately 3650 picoseconds is specific for NADPH bound to oxidative enzymes, that is, mainly to NADPH oxidase. Further evidence for this assumption provided NAD(P)H-FLIM experiments on PMNs performed either at different temperatures (4°C, 20°C, and 37°C) or at different glucose stimulation levels. None of these experiments showed a modification of the fluorescence lifetime of the enzyme-bound NAD(P)H.

### 3.4. Selective Detection of NADPH Oxidase in PMNs Interacting with *Aspergillus Fumigatus*

In Section 3.5, we demonstrate that the fluorescence lifetime corresponding to NADPH bound to NOX2 of approximately 3650 picoseconds, as determined under stimulation of PMNs with PMA, is also observed during the interaction of PMNs with *Aspergillus fumigatus*. In this way, we are able to localise the assembly of NOX2 and, thus, the site of ROS production, in PMNs interacting with *A. fumigatus*.

We performed NAD(P)H-based biexponential FLIM experiments on mixed suspensions of PMNs and aspergillus conidia or hyphae. The time step between two consecutive FLIM series was 60 seconds and each measurement lasted one hour, so that enough phagocytosis events could be observed for the statistics.

**Figure 5:**
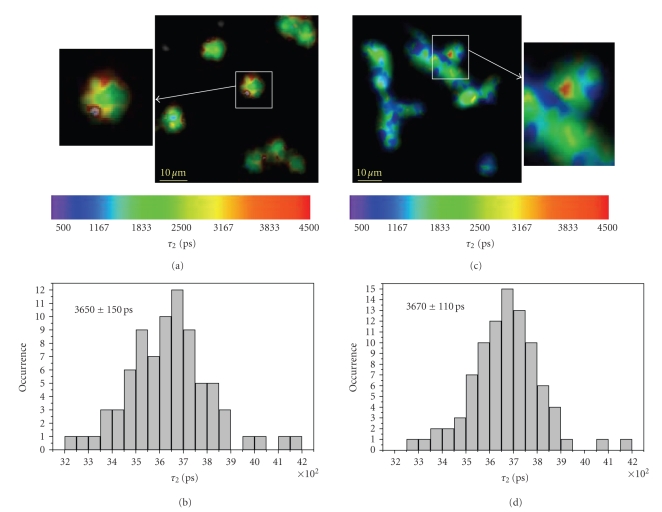
Fluorescence lifetime images of enzyme-bound NAD(P)H (*τ*_2_ images) in PMNs interacting with aspergillus conidia (a) or with two clusters of aspergillus hyphae (c). Also inhere the fluorescence lifetime images were obtained by overlapping intensity images in gray scale with the spatially resolved fluorescence lifetime information in colour scale. Hence both free and phagocytosed conidia in (a) appear in gray tones indicating the absence of enzyme-bound NAD(P)H, whereas PMNs appear in colour due to the fluorescence lifetime of the enzyme-bound NAD(P)H. The increase of the fluorescence lifetime of bound NAD(P)H at the membrane of the phagosome in a PMN (enlarged detail in (a)) can be easily detected in this way. Also at the contact regions between PMNs and aspergillus hyphae (enlarged detail in (c)), the fluorescence lifetime of enzyme-bound NAD(P)H is increased. In order to quantify the increase in fluorescence lifetime of bound NAD(P)H in PMNs interacting with *A. fumigatus*, we performed statistics on the *τ*_2_-distributions of 90 contact regions between PMNs and hyphae and of 80 phagosome membrane regions. The results are depicted in (b) for PMNs interacting with conidia and in (d) for PMNs interacting with hyphae. Note that the average values of both distributions of increased *τ*_2_ amount to approximately 3600 picoseconds and are similar to the value determined in PMNs treated with PMA, confirming the fact that this fluorescence lifetime is specific for NADPH bound to NADPH oxidase. Furthermore, the well-defined sites of this specific lifetime in PMNs indicate the location of ROS production in these cells.

Figures 5(a) and 5(c) exemplary illustrate that the fluorescence lifetime of the enzyme-bound NAD(P)H in PMNs increases after phagocytosis of both conidia and hyphae as compared to the naive cells. Furthermore, they indicate that the regions of increased *τ*_2_ are collocated with the membrane of phagosomes, which contain conidia (Figure 5(a)), and with the contact regions between cells and hyphae (Figure 5(c)), respectively. In these regions, statistics over 80 phagocytosis events of conidia indicate a fluorescence lifetime *τ*_2_ of enzyme-bound NAD(P)H of 3650 ± 140 picoseconds (Figure 5(b)). As far as the phagocytosis of hyphae is concerned, the fluorescence lifetime *τ*_2_ at the contact regions amounts to 3670 ± 110 picoseconds calculated as an average over 90 regions (Figure 5(d)). These values well agree with the lifetime of the NADPH-NOX2 complex measured under stimulation with PMA and confirm the assumption that by means of NAD(P)H-FLIM we are able to observe the activation of oxidative enzymes, especially NADPH oxidase, within cells. The procedure to determine the increased fluorescence lifetime of NADPH bound to oxidative enzymes, that is, mainly NOX2, in PMNs interacting with *A. fumigatus* was the same as that described in Section 3.3 for PMNs treated with PMA.

Neither the fluorescence lifetime *τ*_1_ of free NAD(P)H nor the ratio *a*_2_·*τ*_2_/(*a*_1_·*τ*_1_ + *a*_2_·*τ*_2_) significantly changed in PMNs during or after phagocytosis of *A. fumigatus*.

### 3.5. Dynamics of ROS Production in PMNs

In the previous sections, we demonstrated that a specific fluorescence lifetime of 3650 picoseconds corresponds with high probability to NADPH bound to active NADPH oxidase in selectively stimulated PMNs, and, based on this insight, we located the activation sites of NOX2, that is, the sites of ROS production, in these cells. However, NOX2 activation and ROS production are dynamic processes, which should be characterised in a time-dependent manner. By using multifocal time-gating FLIM, we could easily achieve an acquisition speed of 1 FLIM-frame/minute in NAD(P)H-based fluorescence experiments, which is necessary to observe the dynamics of NOX2 activation in PMNs interacting with aspergillus hyphae.

**Figure 6:**
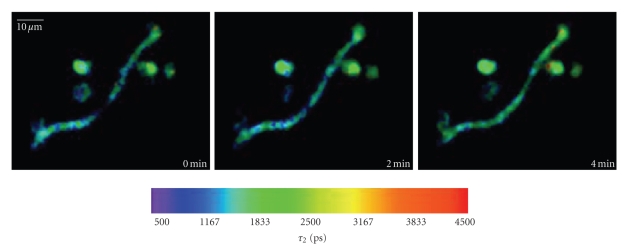
Time series of fluorescence lifetime images of enzyme-bound NAD(P)H (*τ*_2_ images) of PMNs interacting with an aspergillus hypha. Note that the cells which are not in contact with the hypha do not show any increase in fluorescence lifetime, while the PMN which contacts the hypha shows the specific 3600 picoseconds lifetime of NADPH bound to NOX2. The time point 0 was arbitrarily chosen.

While the *τ*_1_-image of free NAD(P)H and the *a*_2_·*τ*_2_/(*a*_1_·*τ*_1_ + *a*_2_·*τ*_2_) ratio image of PMNs interacting with aspergillus hyphae do not show significant changes over time, an increase to approximately 3650 picoseconds is observed in the *τ*_2_-image of enzyme-bound NAD(P)H of the same cells (Figure 6 and supplementary Movie 2). As demonstrated previously, this fluorescence lifetime corresponds to oxidative enzymes, that is, mainly the activated NADPH oxidase, and indicates the production of ROS.

Valuable information provided by the time-lapse FLIM experiments is that oxidative enzymes/NOX2 is activated (ROS are produced) in PMNs interacting with hyphae only at the contact sites and, more important, only during their direct contact (Figure 6). Hence the activation site of the NADPH oxidase is continuously changing within the cell while this is moving along the hypha (supplementary movie 2).

## 4. Discussion

Currently, NAD(P)H-based biexponential fluorescence lifetime imaging is largely employed in monitoring the redox metabolism of living cells in cell culture, tissue, and even organs for bioscientific as well as clinical purposes. Thereby, the free NAD(P)H is distinguished from the NAD(P)H bound to enzymes, which catalyse vital cellular processes, based on its much shorter lifetime (approx. 400 picoseconds) as compared to the lifetime of few nanoseconds for the enzyme-bound NAD(P)H. Although many extracellular studies revealed that the fluorescence lifetime of NAD(P)H strongly depends on the enzyme to which it is bound to, the possibility of selectively detecting enzymes based on the NAD(P)H fluorescence lifetime has not been investigated under intracellular conditions yet. On the example of NAD(P)H-dependent enzymes involved in oxidative processes following phagocytosis, that is, mainly NADPH oxidase in polymorphonuclear cells (neutrophil granulocytes), we demonstrated that NAD(P)H-based FLIM is an advantageous method to localise and to study the function of enzymes intracellularly.

Since it is well known that the fluorescence lifetime of chromophores is significantly influenced by the microenvironment, for proving the selective detection of NADPH oxidase from the pool of mitochondrial and nonmitochondrial enzymes in PMNs based on the fluorescence lifetime of bound NAD(P)H, we performed comparative FLIM experiments on untreated murine PMNs and on PMNs treated with specific activators and inhibitors of NOX2, respectively, rather than matching the lifetime of the isolated NADPH-NOX2 complex measured under extracellular conditions with the spatially resolved fluorescence lifetime of enzyme-bound NAD(P)H in activated PMNs. A further reason for these implicit measurements was the difficult an uncertain activation of NOX2 under cell-free conditions. These studies revealed a typical fluorescence lifetime of approximately 3650 picoseconds mainly located in the membrane regions of PMNs treated with PMA as a specific activator of NOX2. Since neither uninduced murine PMNs nor PMNs treated with inhibitors of NOX2 show such a fluorescence lifetime and neither PMA nor the assembled NOX2 fluoresce under the given experimental conditions, we associated this lifetime value with NADPH bound to oxidative enzymes, that is, mainly NADPH oxidase in PMNs. This assumption was confirmed in FLIM measurements on PMNs interacting with the fungus *Aspergillus fumigatus*. Hence there is strong evidence that the assembled NADPH oxidase can be identified basing on the specific NADPH fluorescence lifetime of 3670±140 picoseconds when bound to this enzyme. Thus we are also able to locate the sites of ROS production in PMNs.

Although time-gated multifocal FLIM is characterised by a rather large signal and background noise, its typical field detection associated with acquisition speedup due to the 64× beam splitting as well as the necessity for relatively few data points on the fluorescence decay curve combined with a noniterative biexponential fitting allowed us to monitor the dynamics of NOX2 assembly and, thus, of ROS production in PMNs interacting with aspergillus hyphae at 1 FLIM-frame/minute. These experiments showed that NOX2 is assembled only at and during the contact between cell and fungus and, thus, the NOX2 activation site continuously changes as the cell is moving along the hypha.

Significantly, higher repetition rates than 1 FLIM-frame/minute can be achieved with the current commercially available devices in experiments based on time-gated multifocal FLIM, if brightly fluorescing chromophores are used. Even for weak-fluorescing chromophores like NAD(P)H, the acquisition speed can be enhanced, if the signal and background noise of the detector (of the MCP intensifier) is reduced. This, however, implies further improvements of the current time-gating devices.

On the example of oxidative enzymes, that is, mainly NADPH oxidase in PMNs, we demonstrated the potential of NAD(P)H-based biexponential fluorescence lifetime imaging to selectively and dynamically identify NAD(P)H- dependent enzymes during their function within living cells. Thus this method opens new ways for fast intravital proteomics, for instance, to a host response to a pathogen, as shown in this study.

## Supplementary Material

Movie 1: CTO stained PMNs (red) interacting with FITC stained conidia (green, small, round) and hyphae (green, ribbon-shaped). The movie is a time series of 3D fluorescence images recorded every 60 seconds Note that the PMNs interact with hyphae both along their length (center) as well as with their conidiophores (right corner), that is, the part of hypha responsible for the asexual reproduction.
Movie 2: The movie is a time series of fluorescence lifetime images of enzyme-bound NAD(P)H of PMNs interacting with an aspergillus hypha recorded every 60 seconds Note that only the PMNs, which contact the hypha shows an increased fluorescence lifetime specific for the NADPH bound to NADPH oxidase. Moreover, this specific fluorescence lifetime appears only during the contact between cell and fungus, meaning that it appears and disappears a few times within the measuring period (30 minutes).
